# Tolerance induced by *Porphyromonas gingivalis* may occur independently of TLR2 and TLR4

**DOI:** 10.1371/journal.pone.0200946

**Published:** 2018-07-24

**Authors:** Wei Lu, Jian-yu Gu, Yao-yao Zhang, Dan-Jun Gong, Yi-ming Zhu, Ying Sun

**Affiliations:** 1 Jiangsu Key Laboratory of Oral Diseases, Nanjing Medical University, Nanjing, China; 2 Department of Periodontology, Affiliated Hospital of Stomatology, Nanjing Medical University, Nanjing, China; 3 Department of Stomatology, Suzhou Hospital, Suzhou, China; University of the Pacific, UNITED STATES

## Abstract

**Objective:**

Periodontitis is a microbe-induced chronic inflammatory disease. Previous exposure of the host to bacteria or their virulence factors leads to refractory responses to further stimuli, which is called tolerance. *Porphyromonas gingivalis* (*P*. *gingivalis*) is one of the most important pathogenic microorganisms associated with periodontitis, and is a potent inducer of pro- and anti-inflammatory cytokines. The aim of this study was to explore the roles and possible mechanisms of tolerance induced by *P*. *gingivalis*.

**Methods:**

THP-1-derived macrophages were pretreated with 1x10^8^ colony-forming units/ml *P*. *gingivalis* ATCC 33277 or 21 clinical isolates from moderate to severe chronic periodontitis patients (24 h), washed (2 h) and treated with *P*. *gingivalis* ATCC 33277 or the same clinical isolates again (24 h). Levels of pro-inflammatory cytokines TNF-α and IL-1β and anti-inflammatory cytokine IL-10 in supernatants were detected by ELISA. Moreover, to identify the possible mechanisms for the changes in cytokine secretion, Toll-like receptor 2 (TLR2) and TLR4 protein expressions were explored in these cells by flow cytometry.

**Results:**

After repeated challenge with *P*. *gingivalis* ATCC 33277 or clinical isolates, production of TNF-α and IL-1β in macrophages was decreased significantly compared with that following a single stimulation (p<0.05), while only comparable levels of IL-10 were detected in *P*. *gingivalis* ATCC 33277 or clinical isolate-tolerized cells (p>0.05). In addition, there was interstrain variability in the ability to induce IL-1β and IL-10 production after repeated *P*. *gingivalis* stimulation. However, no significant changes in TLR2 or TLR4 were detected in macrophages that were repeatedly treated with *P*. *gingivalis* ATCC 33277 or clinical isolates compared with those stimulated with *P*. *gingivalis* only once (p>0.05).

**Conclusions:**

Repeated *P*. *gingivalis* stimulation triggered tolerance, which might contribute to limiting periodontal inflammation. However, tolerance induced by *P*. *gingivalis* might develop independently of TLR2 and TLR4 and be related to molecules in signaling pathways downstream of TLR2 and TLR4.

## Introduction

Periodontitis is a chronic inflammatory disease induced by bacteria and is one of the most common oral diseases in humans. It is characterized by a constant interaction of pathogenic microorganisms in subgingival plaque and host defense systems [1]. Appropriate immune responses of the host are crucial to resistance of invading periodontal pathogens, but overactivated and uncontrolled immune responses can also lead to the destruction of periodontal supporting tissues and tooth loss [2].

*Porphyromonas gingivalis* (*P*. *gingivalis*) is a black-pigmented, Gram-negative bacterium and is thought to be one of the most critical periodontopathic bacteria [3]. There are many virulence factors produced by *P*. *gingivalis*, which are related to the activation of the host immune system, such as lipopolysaccharide (LPS), arginine- and lysine-specific gingipains and collagenase. Biological characteristics of different virulence factors are believed to be dissimilar [4]. In addition, it is well accepted that heterogeneity of virulence among *P*. *gingivalis* strains does exist [5]. However, the exact pathogenicity of different *P*. *gingivalis* strains still needs to be fully elucidated.

Endotoxin tolerance is a hyporesponsiveness of the host to further challenge following a previous exposure to periodontal pathogens or their virulence factors, which leads to the decreased production of some pro-inflammatory cytokines, such as TNF-α and IL-6 [6]. Refractory responses to a subsequent challenge with the same stimuli (homotolerance) are usually stronger than those with different ones (heterotolerance) [7]. Both of these kinds of tolerances contribute to selective reprogramming, the aim of which is to limit inflammatory damage resulting from activation of the immune system. However, this reprogramming favors further infections at the same time [8, 9]. Persistent periodontopathic bacterial stimulation could lead to the development of tolerance in periodontal tissues, which might be very important in maintaining homeostasis. However, the effects and mechanisms of endotoxin tolerance in the development of periodontitis remain unclear.

Toll-like receptors (TLRs) comprise a family of type I transmembrane proteins characterized by an extracellular domain with leucine-rich repeats and a cytoplasmic domain with homology to the IL-1 receptor. Currently, at least 11 different TLRs have been identified in humans [10]. Two of these family members, TLR2 and TLR4 are the principal pattern recognition receptors and signaling molecules in response to virulence factors of *P*.*gingivalis*, such as LPS, fimbriae, peptidoglycan and outer membrane vesicles, and play very important roles in inflammatory responses in periodontal tissues [11, 12].

In this study, we hypothesized that there might be interstrain variabilities in the biological effects due to heterogeneity of virulence among strains of *P*. *gingivalis*. Therefore, we explored the effects of tolerance induced by different *P*. *gingivalis* clinical isolate on cytokine production in macrophages and the possible involvement of TLR2 and TLR4.

## Materials and methods

### Reagents and bacterial strains

THP-1 cells were purchased from Shanghai Institutes for Biological Sciences, Chinese Academy of Sciences (Shanghai, China). *P*. *gingivalis* ATCC 33277 and *E*. *coli* ATCC 25922 were kindly provided by Jiangsu Key Laboratory of Oral Diseases (Nanjing, China). Phorbol-12-myristate-13-acetate (PMA) was obtained from Sigma-Aldrich (Missouri, USA). ELISA kits were provided by Biosource (CO, USA). FITC-conjugated anti-TLR2 antibodies, FITC-conjugated IgG2b isotype control, PE-conjugated anti-TLR4 antibodies and PE-conjugated IgG1 isotype control were from eBioscience (CA, USA).

### Isolation and identification of *P*. *gingivalis* clinical isolates

The Ethical Committee of Nanjing Medical University approved the protocol of this study (Permit Number: 20140304) and written informed consent was obtained from all subjects.

21 Chinese adults with chronic periodontitis (33–62 years old, mean 48.67 years; 11 males and 10 females) who had been referred to the Affiliated Hospital of Stomatology, Nanjing Medical University, Nanjing, China, from July 2014 to May 2015, were enrolled in this study. The inclusion criteria were 1) moderate to severe chronic periodontitis patients with probing depth (PD) ≥5.0 mm, clinical attachment loss (CAL) ≥3.0 mm and alveolar bone resorption exceeding 1/3 of the root in more than 30% of teeth; 2) no systemic diseases, such as diabetes mellitus, cardiovascular diseases and diseases of immune system; 3) no previous periodontal treatment; 4) no use of any immunosuppressive agents, antibiotics or anti-inflammatory drugs in the last 6 months; and 5) no history of smoking.

Before sampling, clinical periodontal parameters, including plaque index (PLI), gingival index (GI), PD and CAL, were recorded and are presented in Table 1. All examinations were made by a calibrated examiner.

**Table 1:** Clinical periodontal parameters of chronic periodontitis patients.

parameters	chronic periodontitis patients
PLI	1.92±0.30
GI	1.72±0.28
PD (mm)	5.85±0.70
CAL (mm)	4.25±1.41

Clinical periodontal parameters of the sampled teeth, including plaque index (PLI), gingival index (GI), probing depth (PD) and clinical attachment loss (CAL), were recorded prior to sampling. The data of PLI, GI, PD and CAL are presented as the mean±standard deviation (SD).

Teeth with PD ≥5.0 mm and bleeding on probing (+) were chosen in each patient. Supragingival calculus and plaque were gently removed and subgingival plaque samples were taken from deep pockets with sterile endodontic paper point as described by Gonçalves [13]. Samples were immediately placed in a vial containing 1 ml of TD transport medium on ice and transferred to the laboratory within 30 min. Then, the freshly collected samples were diluted, plated out on selective culture medium for bacteroid (brain heart infusion agar plates containing 5% sheep blood, 5 mg/l hemin, 1 mg/l menadione, 75 μg/ml kanamycin and 2 μg/ml vancomycin), and grown for 7–10 days anaerobically (75% N_2_, 10% CO_2_, 15% H_2_) at 37°C. Bacteria were selected on the basis of the appearance of colonies and Gram staining. Gram-negative rods with black-pigmented colonies were purified by subculture and submitted to PCR with 16S rRNA-based *P*. *gingivalis*-specific primers [14] and sequencing of products (S1 Fig). A total of 21 isolates identified as *P*. *gingivalis* were kept at -70°C for subsequent experiments.

### Cell culture and *P*. *gingivalis* stimulation

THP-1 cells were seeded in 6-well plates (5x10^5^ cells/ml) and cultured in RPMI 1640 medium with 100 nM PMA for 48 h. After differentiation, nonattached cells were aspirated, and the remaining adherent macrophages were washed with fresh RPMI 1640 medium three times. Then, the PMA-differentiated THP-1 cells were divided into 7 groups. Cells of group A were only stimulated with culture medium. Groups B, D and F were cultured in medium for 24 h, washed three times and challenged with 1x10^8^ CFU/ml heat-inactivated *P*. *gingivalis* clinical isolates, *P*. *gingivalis* ATCC 33277 or *E*. *coli* ATCC 25922 (served as positive controls), respectively, for 24 h. Cells of groups C, E and G were treated with 1x10^8^ CFU/ml *P*. *gingivalis* clinical isolates, *P*. *gingivalis* ATCC 33277 or *E*. *coli* ATCC 25922 for 24 h, washed, and retreated with the same stimulation for another 24 h.

### TNF-α, IL-1β and IL-10 detection

After the above-mentioned stimulation, cell free supernatants were collected and kept at -40°C for the following cytokine assays. Levels of TNF-α, IL-1β and IL-10 in the supernatants were measured by ELISA, and the absorbance was detected in a microplate reader (Bio-Hit, Helsinki, Finland) at 490 nm.

### TLR2 and TLR4 expression levels determination

Macrophages were scraped off 6-well plates and washed three times with PBS containing 1% fetal calf serum (FCS). To explore the surface expression of TLR2 and TLR4, cells were stained with 1 μg of FITC-conjugated anti-TLR2 antibodies and 1 μg of PE-conjugated anti-TLR4 antibodies/10^6^ cells in 100 μl FCS buffer at 4°C in the dark. FITC-conjugated IgG2b isotype and PE-conjugated IgG1 isotype were employed as controls for nonspecific binding of the above-mentioned antibodies. After 30 min, cells were washed twice and then fixed in 1% formalin for an additional 30 min at 4°C. Viable cells (10,000 per sample) were then examined using a FACSCalibur flow cytometer (BD Biosciences, USA).

### Statistical analysis

The ELISA data were analyzed by ANOVA, and differences between groups were compared by Dunnett's T3 test. Statistical analysis of flow cytometry data was performed using the Kruskal–Wallis test. The Mann-Whitney test was then used as a post-hoc test. All data are expressed as the means ± SD and considered significant at p<0.05.

## Results

### Cytokine production in macrophages upon a primary or secondary exposure to *P*. *gingivalis*

**Fig 1:**
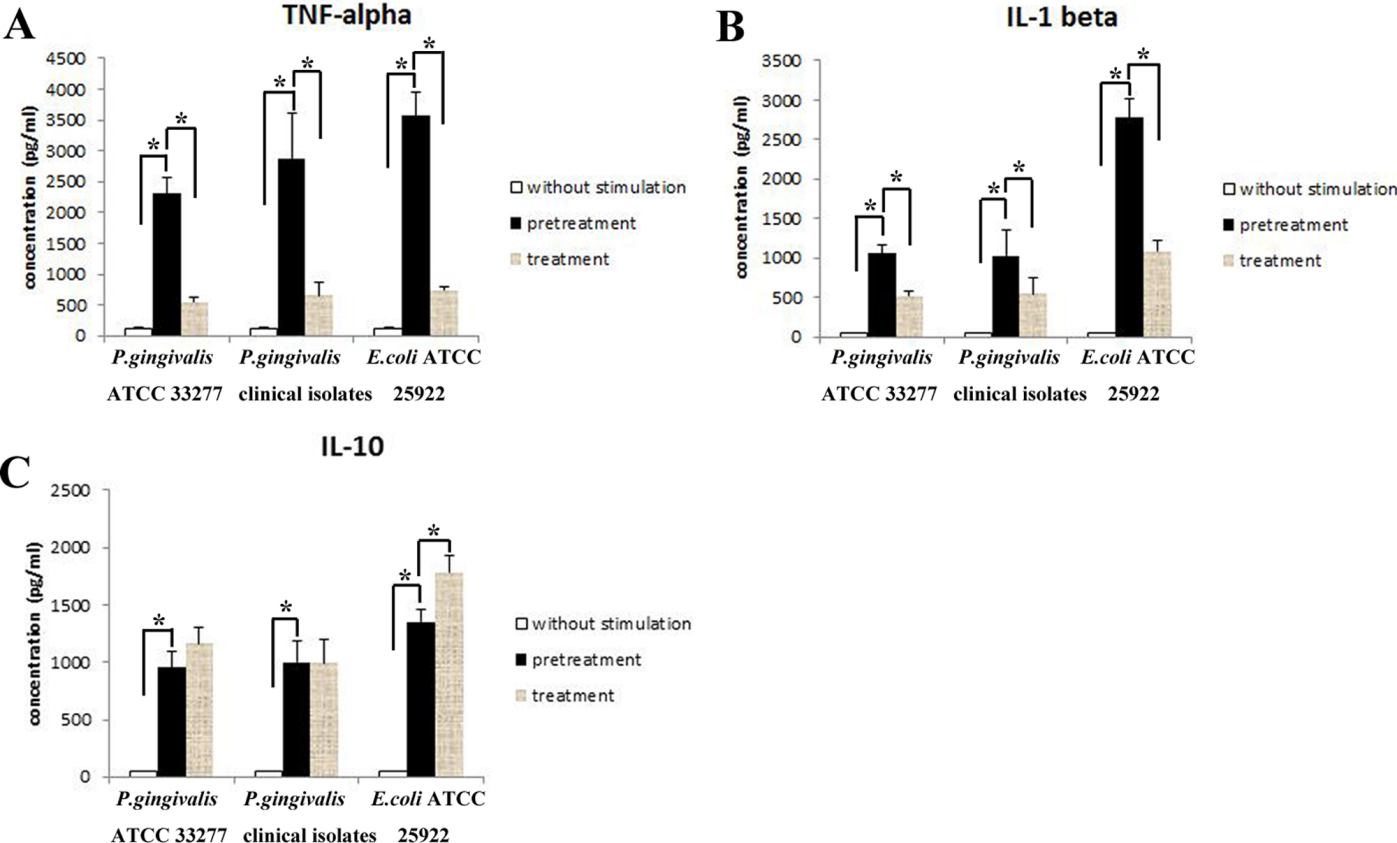
Cytokine expression in macrophages stimulated with *P*. *gingivalis*. Macrophages were stimulated with medium, 1x10^8^ CFU/ml heat-inactivated *P*. *gingivalis* clinical isolates, *P*. *gingivalis* ATCC 33277 or *E*. *coli* ATCC 25922 (positive control) for 24 h, washed and then challenged with medium, 1x10^8^ CFU/ml *P*. *gingivalis* clinical isolates, *P*. *gingivalis* ATCC 33277 or *E*. *coli* ATCC 25922 for another 24 h. Expression levels of TNF-α (A), IL-1β (B) and IL-10 (C) in the cell-free supernatants were detected by ELISA. Data are presented as the mean±SD (n = 5 per group). *p<0.05.

We first performed ELISA assays to explore the secretions of the pro-inflammatory cytokines TNF-α and IL-1β and anti-inflammatory cytokine IL-10 by PMA-differentiated macrophages. Our results revealed that the amounts of TNF-α and IL-1β secreted by macrophages stimulated with *P*. *gingivalis* clinical isolates, *P*. *gingivalis* ATCC 33277 and *E*. *coli* ATCC 25922 were increased significantly compared with the cells without stimulation (p<0.05). In addition, the levels of TNF-α secreted by the cells with *E*. *coli* ATCC 25922 stimulation were higher than those with *P*. *gingivalis* ATCC 33277 stimulation (p<0.05), while there were no significant differences in the cells stimulated with *P*. *gingivalis* clinical isolates and *E*. *coli* ATCC 25922 (p>0.05). However, release of IL-1β in macrophages stimulated with *E*. *coli* ATCC 25922 was significantly higher than that in macrophages stimulated with *P*. *gingivalis* ATCC 33277 and clinical isolates (p<0.05). In addition, there were also no significant differences in the production of TNF-α and IL-1β in the cells stimulated with *P*. *gingivalis* clinical isolates and *P*. *gingivalis* ATCC 33277 (p>0.05). After restimulation with the same bacteria for another 24 h, TNF-α and IL-1β levels were decreased significantly compared with those with only a single challenge (*P*. *gingivalis* clinical isolates, *P*. *gingivalis* ATCC 33277 or *E*. *coli* ATCC 25922 separately) (p<0.05) (Fig 1A).

In addition, the levels of IL-10 were also examined in macrophages with and without bacterial stimulation. After stimulation with *P*. *gingivalis* clinical isolates, *P*. *gingivalis* ATCC 33277 or *E*. *coli* ATCC 25922, production of IL-10 was significantly increased compared with those without stimulation (p<0.05). Moreover, the levels of IL-10 were significantly higher in cells stimulated with *E*. *coli* ATCC 25922 than in those stimulated with *P*. *gingivalis* clinical isolates or *P*. *gingivalis* ATCC 33277 (p<0.05). There were no significant differences in the cells stimulated with *P*. *gingivalis* clinical isolates and those stimulated with *P*. *gingivalis* ATCC 33277 (p>0.05). After repeated bacterial stimulation, the amounts of IL-10 were increased significantly in macrophages restimulated with *E*. *coli* ATCC 25922 (p<0.05) but not in cells restimulated with *P*. *gingivalis* clinical isolates or *P*. *gingivalis* ATCC 33277 (p>0.05) (Fig 1B).

**Fig 2:**
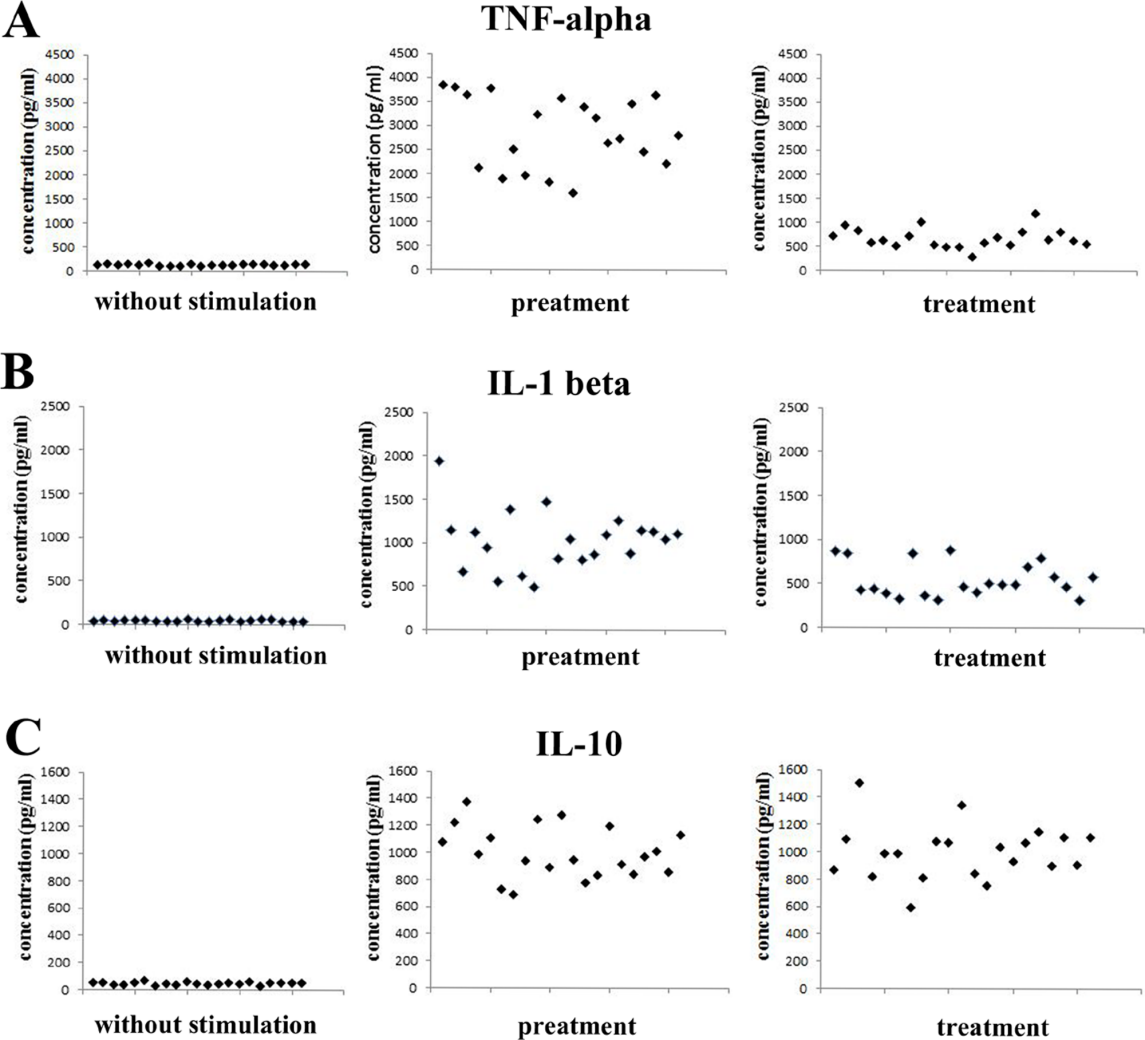
Interstrain variability of cytokine production in macrophages stimulated with *P*. *gingivalis* clinical isolates. Macrophages were incubated with 1x10^8^ CFU/ml heat-inactiveted *P*. *gingivalis* clinical isolates (a total of 21 strains) for 24 h, washed and then restimulated with the same clinical isolates (1x10^8^ CFU/ml) for another 24 h. TNF-α (A), IL-1β (B) and IL-10 (C) levels in the culture supernatants were determined by ELISA.

It was interesting to discover that there were inter-strain variabilities among *P*. *gingivalis* clinical isolates in the ability to induce the production of IL-1β and IL-10 but not TNF-α. There was no significant change in IL-1β secretion in macrophages retreated with 1 strains of clinical isolates (1/21) compared with those challenged with corresponding clinical isolate only once (p<0.05). Moreover, significantly increased IL-10 levels were found in macrophages restimulated with 3 strains of clinical isolates (3/21) compared with those stimulated with corresponding clinical isolates only once (p<0.05) (Fig 2).

### Expression of TLR2 and TLR4 in macrophages after a primary or secondary *P*. *gingivalis* exposure

**Fig 3:**
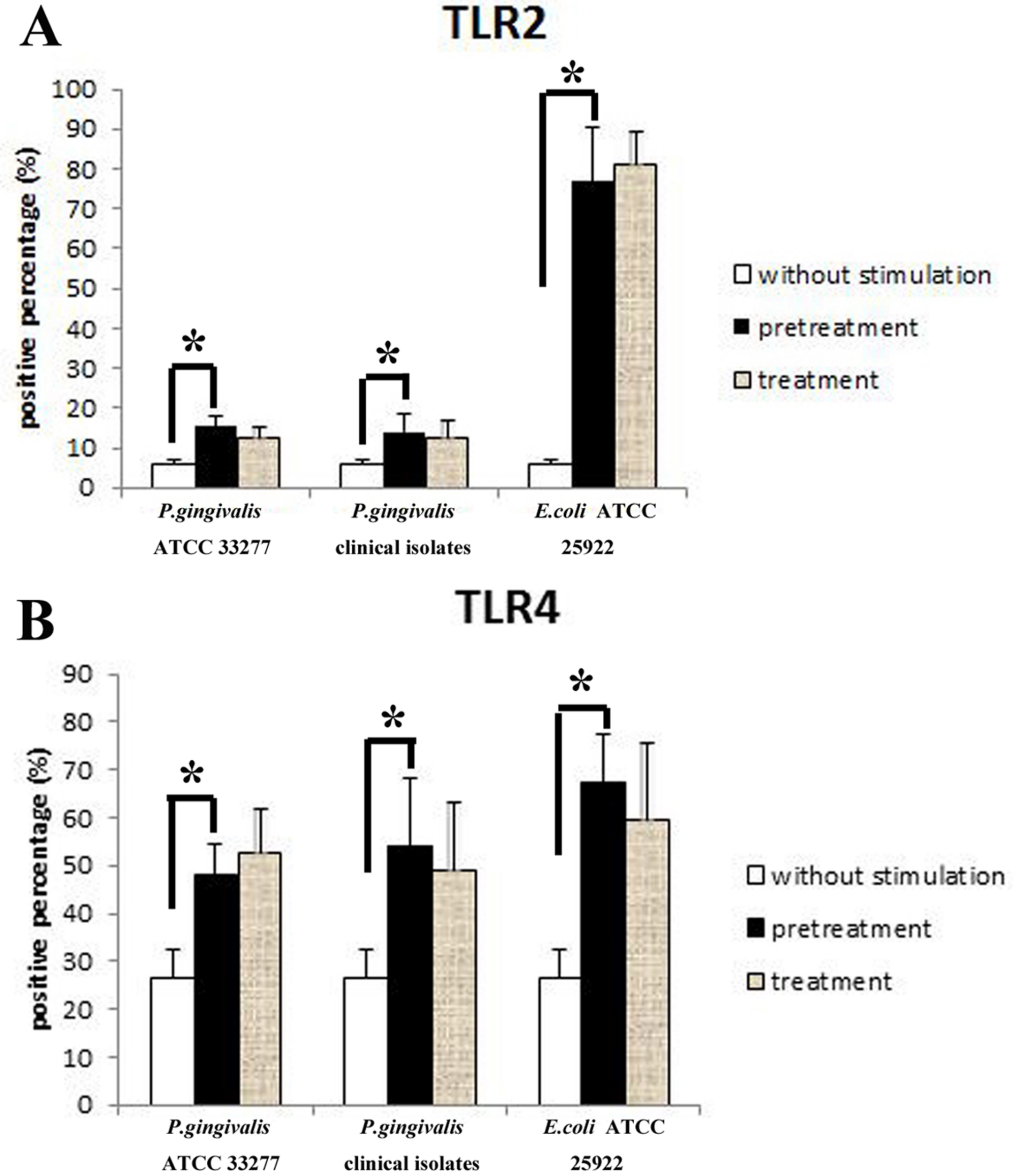
Protein expression of TLR2 and TLR4 in macrophages stimulated with *P*. *gingivalis*. Macrophages were challenged with medium or *P*. *gingivalis* as described in the legend to Fig 1. Flow cytometry was employed to detect the protein expression level of TLR2 (A) and TLR4 (B). Data are presented as the mean±SD (n = 5 per group). *p<0.05.

**Fig 4:**
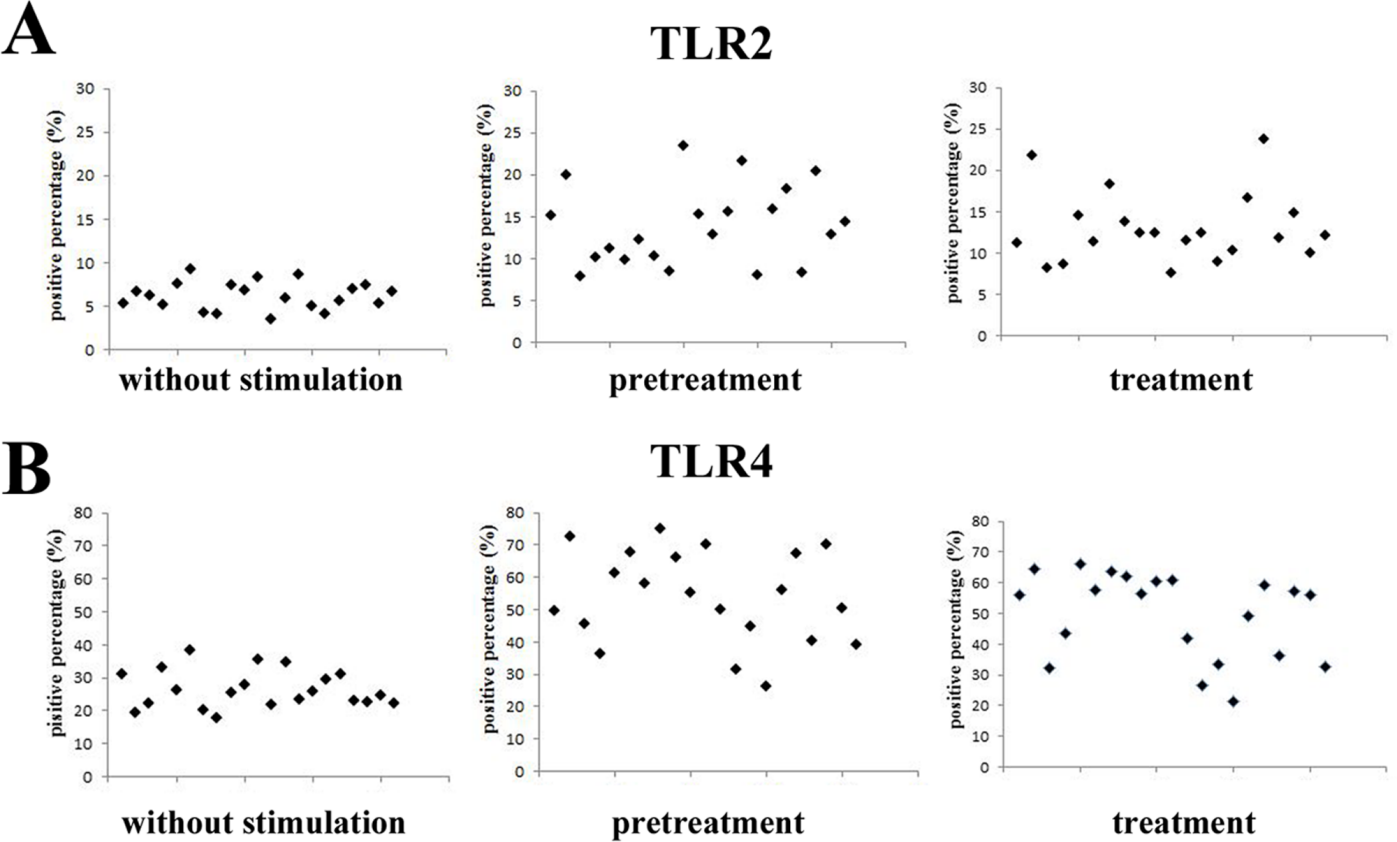
Interstrain variability of TLR2 and TLR4 expression in macrophages stimulated with *P*. *gingivalis* clinical isolates. Macrophages were stimulated with *P*. *gingivalis* as described in the legend to Fig 2. Flow cytometry was used to quantify the expression levels of TLR2 (A) and TLR4 (B).

**Fig 5:**
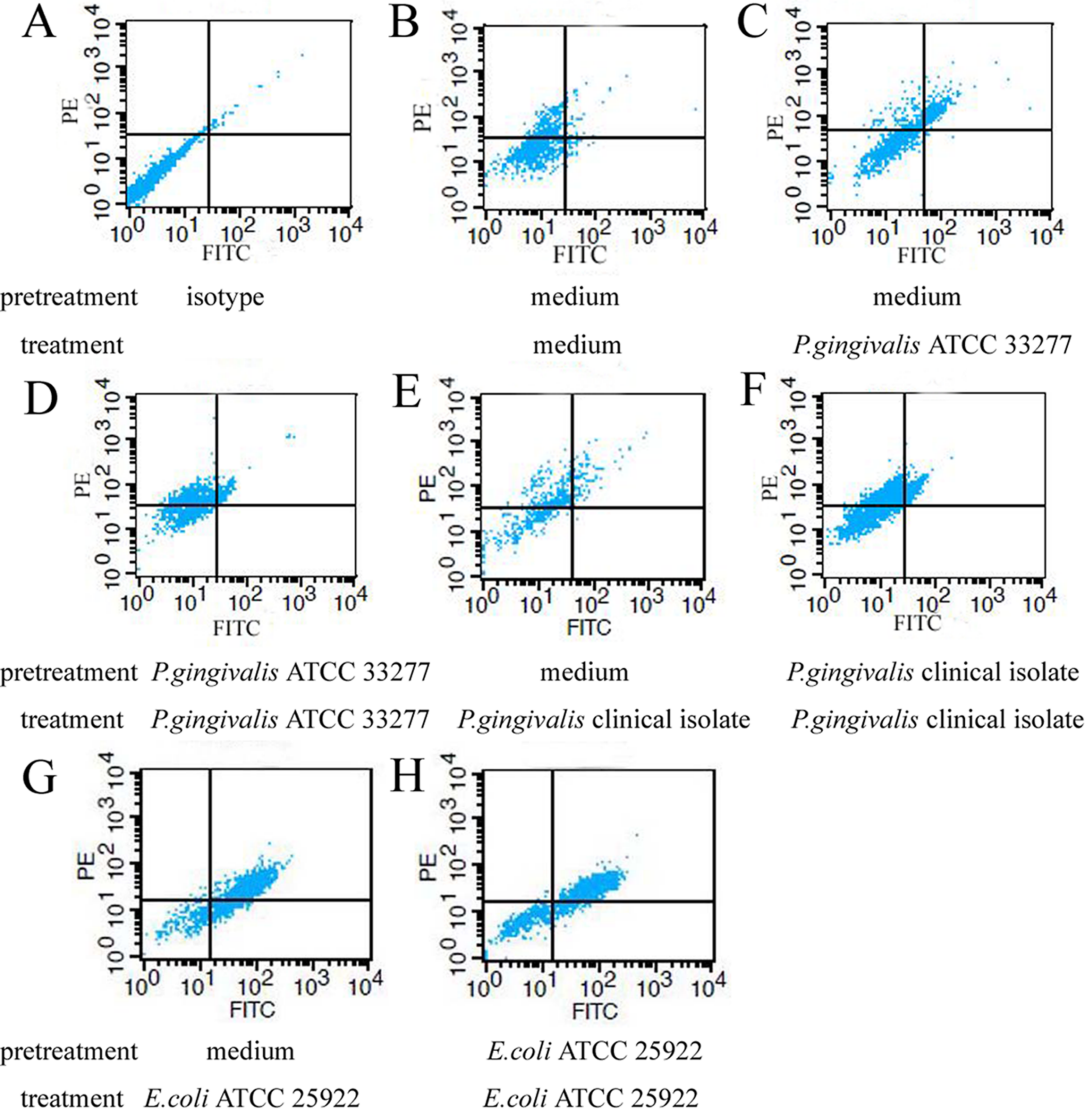
Surface expression of TLR2 and TLR4 in macrophages treated with *P*. *gingivalis* measured by flow cytometry. Macrophages were stimulated with medium or *P*. *gingivalis* as described in the legend to Fig 1. Surface expression of FITC-conjugated TLR2 and PE-conjugated TLR4 were explored by flow cytometry. One representative result of five repeated experiments is shown.

Upregulation of TLR2 was observed in macrophages stimulated with *P*. *gingivalis* ATCC 33277, *P*. *gingivalis* clinical isolates or *E*. *coli* ATCC 25922 (p<0.05). In addition, TLR2 expression levels in PMA-differentiated macrophages with *E*. *coli* treatment were higher than those treated with *P*. *gingivalis* ATCC 33277 or *P*. *gingivalis* clinical isolate stimulation (p<0.05). No significant differences were confirmed between macrophages stimulated with *P*. *gingivalis* clinical isolates and *P*. *gingivalis* ATCC 33277 (p>0.05). Surprisingly, compared with the macrophages challenged with *P*. *gingivalis* clinical isolates, *P*. *gingivalis* ATCC 33277 or *E*. *coli* ATCC 25922 only once, there were no changes in TLR2 protein expression in the cells retreated with the same bacteria (p>0.05) (Figs 3A and 4A). One representative result of five independent flow cytometry detections is shown in Fig 5.

Similar to the changes noted above for TLR2, increased expression of TLR4 was also observed in macrophages stimulated with *P*. *gingivalis* clinical isolates, *P*. *gingivalis* ATCC 33277 or *E*. *coli* ATCC 25922 (p<0.05). Moreover, higher levels of TLR4 were also exhibited in cells stimulated with *E*. *coli* than in those treated with *P*. *gingivalis* ATCC 33277 (p<0.05), but not *P*. *gingivalis* clinical isolates (p>0.05). There were no significant differences in TLR4 expression between macrophages stimulated with *P*. *gingivalis* clinical isolates and *P*. *gingivalis* ATCC 33277 (p>0.05). In addition, compared with the cells stimulated with *P*. *gingivalis* clinical isolates, *P*. *gingivalis* ATCC 33277 or *E*. *coli* ATCC 25922, no significant changes in TLR4 protein expression were observed after the same bacterial restimulation (p>0.05) (Figs 3B, 4B and 5).

Even if there were interstrain variabilities among *P*. *gingivalis* clinical isolates in the ability to activate TLR2 and TLR4, there were no significant differences in TLR2 and TLR4 expression between cells with individual clinical isolate stimulation and restimulation (p>0.05).

## Discussion

In this study, we explored cytokine secretion in PMA-differentiated macrophages stimulated with repeated *P*. *gingivali*s ATCC 33277 or clinical isolates and revealed the potential differences in the ability to induce endotoxin tolerance by different *P*. *gingivali*s clinical strains at the levels of the pro-inflammatory cytokines TNF-α and IL-1β, for the first time. Moreover, even if *P*. *gingivalis* could activate TLR2 and TLR4, tolerance induced by these bacteria might occur independently of the above-mentioned pattern recognition receptors (PRRs).

*P*. *gingivalis* has been proven to be one of the most critical etiological agents in the development of periodontitis, which can lead to the breakdown of periodontal tissue and the loss of teeth [15, 16]. A survey in an American population indicated that *P*. *gingivalis* was detected in 25% of the healthy subjects and 79% of the periodontitis group [17]. Another study conducted in Chinese patients also disclosed that *P*. *gingivalis* was found in 86.7% of the healthy sites and 95% of the inflammatory sites (PD 4–6 mm) in periodontitis patients, while in only 27.4% of sulci in healthy controls [18], implying that virulence of *P*. *gingivalis* in inflammatory sites of periodontitis patients might be stronger than those in healthy sites/persons and more likely to induce severe infection in the future.

Compared with *E*. *coli*, the classic Gram-negative bacteria, *P*. *gingivalis* is an unusual pathogen involved in commensal periodontal microbiota. One of the most important virulence factors of *P*. *gingivalis*, LPS, is a special PRR ligand in the innate immune system. Compared with *E*. *coli* LPS, its endotoxic activity is very low. It is both agonist and antagonist for TLR4 depending on hemin concentrations in the microenvironment [19]. Moreover, *P*. *gingivalis* attenuated the production of IL-8 and intercellular adhesion molecule-1 (ICAM-1) in epithelial cells and blocked neutrophil migration across intact epithelial barriers, which impairs the ability of the host to detect bacteria and contributes to sustained bacterial infection [20].

Accumulating evidence indicates that heterogeneity in virulence exists among different *P*. *gingivalis* strains, as assayed in experimental model systems [21, 22]. There are several kinds of classifications for *P*. *gingivalis* subtypes according to the capsule, serum type or fimbria gene. In early studies, encapsulated cells, such as W50, were identified as virulent or invasive strains, which could induce phlegmonous abscess, necrosis, and even death in experimental animals, while nonencapsulated cells, such as ATCC 33277, were classified as nonvirulent or noninvasive strains, which could only lead to localized abscesses [23]. Van Winkelholf examined the prevalence and distribution of six K-typeable strains in periodontitis patients and disclosed that K5 and K6 subtypes were more common in periodontal infections [24]. In addition, on the basis of the nucleotide sequences of the fimA gene, strains of *P*. *gingivalis* can also be classified into six variants (types I to V and I b). Among them, virulence of type II is the strongest and can firmly adhere to host proteins [25]. In this study, we chose *P*. *gingivalis* ATCC 33277, which is a low toxicity strain and more common in humans, as a control.

Production of pro-inflammatory cytokines, including TNF-α and IL-1β, and anti-inflammatory cytokines, such as IL-10, is one of the most essential responses of the host to invading periodontal pathogens. A balance of pro-inflammatory cytokines and anti-inflammatory cytokines is critical for homeostasis in the host. Then, an appropriate innate immune response will be developed to resist periodontopathic bacteria without excessive damage to periodontal tissues. Numerous studies revealed the increased production of TNF-α, IL-1β and IL-10 in monocytes/macrophages upon stimulations of *P*. *gingivalis* or its virulence factors [26–29]. However, differences in biological effects might exist between the whole cell of *P*. *gingivalis* and its single virulence factors, such as LPS and FimA. Zhou indicated that purified *P*. *gingivalis* LPS and FimA induced similar cytokine profiles in macrophages, while cytokine patterns induced by *P*. *gingivalis* were significantly different. His study also reported that the expression of proteins involved in gene transcription, signal transduction, immune responses and apoptosis were modulated differentially by *P*. *gingivalis*, its LPS and FimA [30]. Moreover, interstrain variability among *P*. *gingivalis* strains in the ability to induce the production of various cytokines, such IL-1β, IL-8 and IL-10, were observed by numerous researchers [31, 32]. On the basis of our previous researches concerning endotoxin tolerance induced by *P*. *gingivalis* LPS [27, 29], this study revealed differences in TNF-α, IL-1β and IL-10 secretions in macrophages treated with different *P*. *gingivalis* isolates, which were consistent with the above-mentioned articles [30–32]. Interestingly, Baek’s study indicated that even if TNF-α and IL-1α were less susceptible to degradation, differential degradation of cytokines (including IFN-γ and IL-17A) by *P*. *gingivalis* did exist [32], which might have an effect on the expression levels of cytokines in cells treated with *P*. *gingivalis*.

In fact, endotoxin tolerance is not an overall decrease of all cytokines. In contrast, it represents a selective reprogramming of gene and protein expression [29]. TNF-α might be an excellent marker for endotoxin tolerance, and many researchers found decreased production of TNF-α in in vivo and in vitro endotoxin tolerance models [33, 34]. Similar changes in IL-6 and opposite changes in IL-10 were revealed by other studies in early or late stages [35, 36]. Previous research from our group also confirmed decreased secretions of TNF-α and IL-1β, increased levels of IL-10 and comparable production of IL-8 in THP-1 cells with repeated *P*. *gingivalis* LPS or *E*. *coli* LPS stimulation [29]. Consistent with the above results, similar changes in TNF-α, IL-1β and IL-10 in macrophages treated with repeated *E*. *coli* stimulation were also demonstrated in this study. However, tolerance triggered by both *P*. *gingivalis* ATCC 33277 and clinical isolates could only lead to lower expression levels of TNF-α and IL-1β, but not higher levels of IL-10, which might be related to the differences of biological activities and pathogenicity between *E*. *coli* and *P*. *gingivalis*.

TNF-α and IL-1β are pro-inflammatory cytokines involved in inflammation and regulation of immune cells. Their downregulated production in tolerance induced by *P*. *gingivalis* might contribute to the limitation of immune damage in periodontal tissues. IL-10 is an anti-inflammatory cytokine with multiple effects in immunoregulation and inflammation, such as downregulation of Th1 cytokines, MHC class II antigens and costimulatory molecules and is involved in the regulation of the JAK-STAT signaling pathway. STAT3 is a transcription factor modulating anti-inflammatory properties of IL-10. *P*. *gingivalis* LPS could down-regulate STAT3 expression in macrophages, while FimA has a contrary effect. Therefore, levels of STAT3 could not be changed by whole bacterial stimulation [30], which might be related to the differences in IL-10 secretion in tolerized macrophages triggered by the *P*. *gingivalis* strain and its LPS. Un-changed production of IL-10 in *P*. *gingivalis*-tolerized macrophages might favor killing invading pathogens, while having no effect on controlling immune damage.

It has been demonstrated that TLR2 and TLR4 might be responsible for recognizing pathogen-associated molecular patterns from *P*. *gingivalis* and be involved in their signal transduction. Su’s study revealed that *P*. *gingivalis* LPS triggered DCs through the TLR2, but not the TLR4 pathway [37], while Herath suggested that tetra- and penta-acylated lipid A structures of *P*. *gingivalis* LPS differentially activate the TLR4-mediated NF-κB signaling pathway and modulate IL-6 and IL-8 production in human gingival fibroblasts [38]. Moreover, research concerning TLR2- and TLR4-deficient macrophages revealed that most of the cytokine production induced by *P*. *gingivalis* LPS or FimA was through TLR2-mediated signaling pathway, while most of the cytokine secretion induced by the *P*. *gingivalis* strain was through both TLR2- and TLR4-mediated signaling pathway, which agreed with our results [39].

Some previous studies disclosed the involvement of TLR2 and/or TLR4 in endotoxin tolerance, while others disagreed [40–42]. In our present study, even if *P*. *gingivalis* stimulation increased TLR2 and TLR4 expression, there were no changes in TLR2 and TLR4 levels in *P*. *gingivalis*-tolerized macrophages. Medvedev confirmed the importance of TLR4 tyrosine phosphorylation for signaling and speculated that it might be impaired in LPS-tolerized cells. In addition, he also suggested that induction of endotoxin tolerance could also markedly suppress LPS-mediated recruitment of Lyn kinase to TLR4 [43]. It was expression levels of TLR2 and TLR4, but not their activities, that were explored in this research. Therefore, activities of TLR2 and TLR4, such as their tyrosine phosphorylation, should be investigated in the future.

Moreover, there were many virulence factors in *P*. *gingivalis*, such as gingipains and peptidoglycan. Tolerance induced by *P*. *gingivalis* might be heterotolerance rather than homotolerance. Effects and signal transduction pathways of heterotolerance triggered by different LPS might be more complex than those of homotolerance. Cole’s research indicated that IRAK-M played an important role in release of IL-10 during heterotolerance induced by LPS and *Helicobacter pylori* sonicate [44]. Dobrovolskaia found that heterotolerance was weaker than TLR2 or TLR4 homotolerance with the exception of IKK kinase activity. In addition, TNF-α secretion was suppressed in *P*. *gingivalis* LPS-pretreated and *E*. *coli* LPS-challenged cells, but not vice versa [7]. Synergy between TLR2- and TLR4-mediated signaling pathways might exist. Sato found that there were severely impaired NF-κB and c-Jun NH(2)-terminal kinase but not decreased surface expression of TLR4 in macrophage-activating lipopeptides (MALP-2)-pretreated and LPS stimulated cells. Then, he speculated that heterotolerance between MALP-2 and LPS might be related to regulation of downstream cytoplasmic signaling pathways [45]. Therefore, we reasoned that there might be some other signal molecules involved in *P*. *gingivalis*-induced tolerance, which needs to be explored further.

In summary, this study revealed the suppressed production of TNF-α and IL-1β and comparable secretion of IL-10 in *P*. *gingivalis*-tolerized macrophages, which might not only contribute to the limitation of inflammation and immune damage but also harm killing invading bacteria. Moreover, tolerance induced by *P*. *gingivalis* was not related to the expression levels of TLR2 and TLR4. Further investigations are necessary for a better understanding of its molecular mechanisms.

## Supporting information

S1 Fig Identification of *P*. *gingivalis* clinical isolates. Subgingival plaque samples were collected from adults with untreated chronic periodontitis and cultured on selective culture medium for bacteroid for 7–10 days anaerobically. According to the appearance of colonies (A), Gram staining (B), PCR (C) and sequencing of PCR products (D). *P*. *gingivalis* were isolated and identified for the following experiments.
(TIF)
