# Supplementary figures and images for: Tolerance induced by Porphyromonas gingivalis may occur independently of TLR2 and TLR4
Source: PLoS One. 2018 Jul 24;13(7):e0200946. doi: 10.1371/journal.pone.0200946 (PMC6057631; doi:10.1371/journal.pone.0200946)

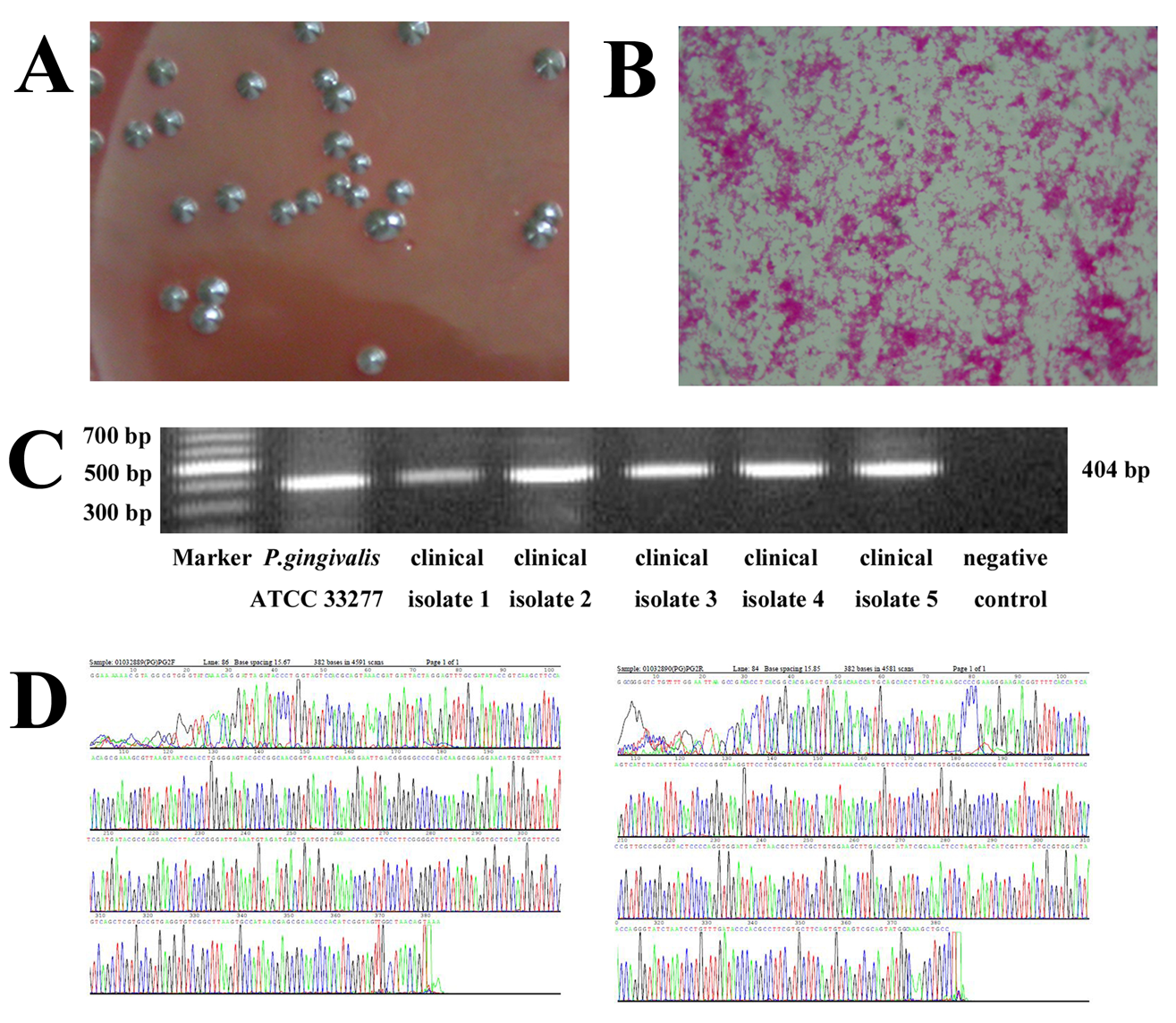

Supplement: S1 Fig — Subgingival plaque samples were collected from adults with untreated chronic periodontitis and cultured on selective culture medium for bacteroid for 7–10 days anaerobically. According to the appearance of colonies (A), Gram staining (B), PCR (C) and sequencing of PCR products (D). P. gingivalis were isolated and identified for the following experiments. (TIF) [file pone.0200946.s001.tif]
